# The “eye sign” due to hemispatial neglect: A case report

**DOI:** 10.1590/S1980-57642009DN30300013

**Published:** 2009

**Authors:** Fábio Henrique de Gobbi Porto, Gislaine Cristina Lopes Machado, Mari-Nilva Maia da Silva, Gabriel Rodriguez de Freitas

**Affiliations:** 1 MD, Behavioral and Cognitive Neurology Unit, Department of Neurology, and Cognitive Disorders Reference Center (CEREDIC), Hospital das Clínicas of the University of São Paulo, São Paulo, SP, Brazil.; 2 MD. Department of Radiology, Hospital A.C. Camargo, São Paulo, SP, Brazil.; 3 MD, Behavioral and Cognitive Neurology Unit, Department of Neurology, and Cognitive Disorders Reference Center (CEREDIC), Hospital das Clínicas of the University of São Paulo, São Paulo, SP, Brazil.; 4 MD, PhD, Cerebrovascular Unit, Department of Neurology, Hospital Antônio Pedro of the Federal Fluminense University (UFF), Rio de Janeiro, RJ, Brazil.

**Keywords:** Hemispatial neglect, insular stroke, conjugate eye deviation, “eye sign”

## Abstract

Conjugate eye deviation is characterized by a sustained shift in horizontal gaze, usually toward the affected brain hemisphere. When detected on neuroimaging, it is called the “eye sign”. It is classically associated with lesions involving the frontal eye fields, ipsilateral to the side of the deviation. Neglect may be conceptualized as a spatially addressed bias of the sensory events in explicit behaviors and in the absence of perceptual and motor deficits. Hemispatial neglect is a common disabling condition that occurs following acute unilateral brain damage, usually to the right side. We report a case of a patient presenting with the “eye sign” on tomography, following an acute subinsular stroke, in the absence of conjugated eyes deviation. Our hypothesis was that the sign may have been due to hemispatial neglect in this patient. The aim of this article was to discuss the mechanisms involved in the attention network and its neuroanatomic correlates.

## Abstract (pt)

Desvio conjugado do olhar é caracterizado por um desvio sustentado horizontal da mirada, normalmente para o lado do hemisfério afetado. Quando visto por métodos de imagem, é chamado “sinal do olhar”. O sinal é normalmente associado a lesões envolvendo o “campo frontal do olho” ipsilateral ao lado do desvio. Negligência pode ser conceitualizada como um viés espacial dos eventos sensoriais no comportamento explícito, na ausência de alterações da percepção e motoras. Negligência hemiespacial é uma condição comum e incapacitante que ocorre após lesão cerebral aguda, principalmente à direita. Nós relatamos um caso de um paciente que apresenta o “sinal do olhar” na tomografia, após um infarto subinsular, na ausência de desvio conjugado do olhar. Nossa hipótese é que, neste paciente, o sinal pode ser devido à negligência hemiespacial. O objetivo deste artigo é discutir os mecanismos envolvidos na rede da atenção e seus correlatos anatômicos.

Conjugate eye deviation is characterized by a sustained shift in horizontal gaze, usually toward the affected brain hemisphere. The deviation can, in some cases, be corrected by oculocephalic maneuvers^1^. When detected on neuroimaging, it is called the “eye sign”.^2^ This phenomenon is also known by its eponymous; Prévost’s sign^3^ and is traditionally associated with lesions involving the frontal eye fields, ipsilateral to the side of the deviation. Parietal lobe, thalamic and internal capsule lesions have also been implicated in conjugate eye deviation.^4^ The condition has been associated with a high value in determining the affected hemisphere^1^ and to a poor prognostic in stroke patients.^5,6^

Neglect may be conceptualized as a spatially addressed bias of the sensory events in explicit behaviors and in the absence of perceptual and motor deficits.^7^ Hemispatial neglect is a common disabling condition that occurs following acute unilateral brain damage, usually to the right side. In the acute care setting, some form of neglect is present in up to two thirds of right hemisphere stroke cases.^8^

We report a case of a patient presenting with the “eye sign” on tomography, following an acute subinsular stroke and in the absence of conjugated eye deviation. Our hypothesis was that the sign may have been due to hemispatial neglect in this patient. The aim of this article was to discuss the mechanisms involved in the attention network and its neuroanatomic correlates.

## Case report

**Figure 1:**
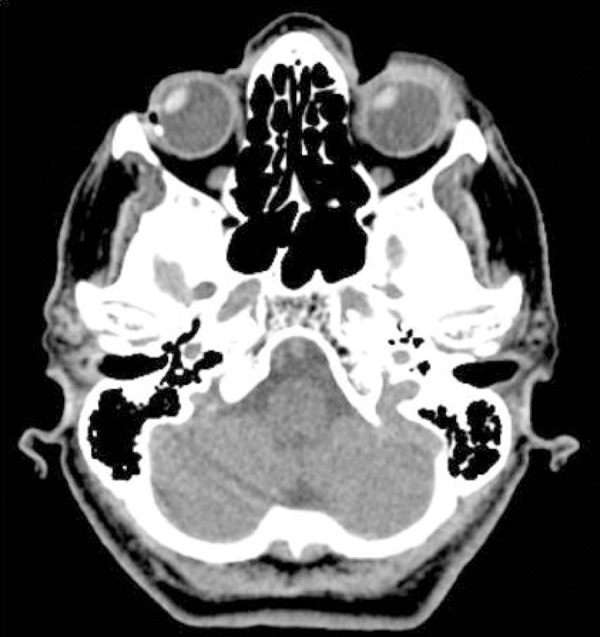
Non-contrast computed tomography (CT) scan showing conjugate eye deviation to the right side.

**Figure 2:**
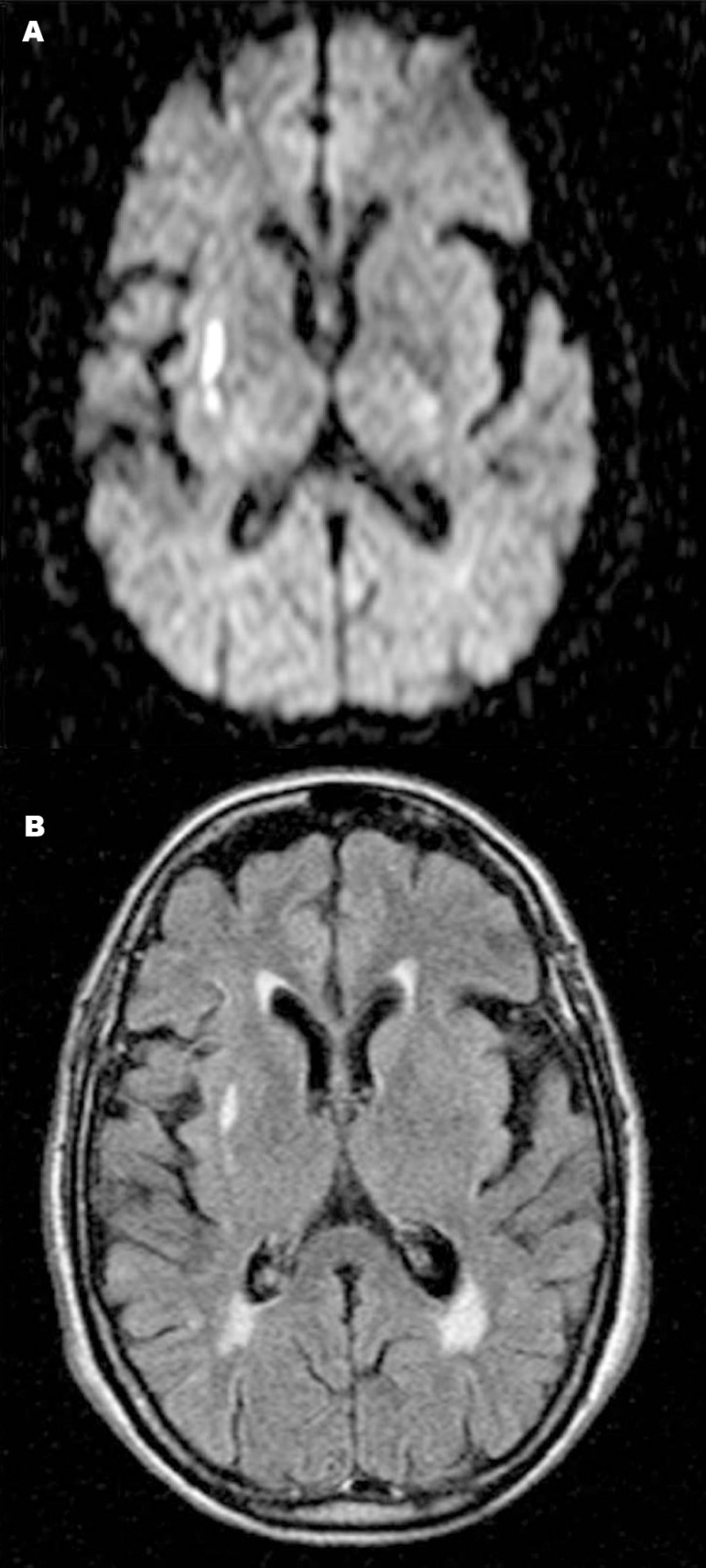
Axial diffusion-weighted magnetic resonance image [A] and [B]; FLAIR MRI [B] demonstrating high signal intensity in the right subcortical insular region.

An 83-year-old right-handed woman presented at the emergency room reporting a 90-minute history of sluggish speech and left side weakness. Her past medical history was marked by hypertension and hypercholesterolemia. The initial neurologic examination showed left side facio-brachio-crural paresis, left hypoesthesia, dysarthria and left side extinction on double tactile and visual stimulation. Approximately 20 minutes later, she developed massive neglect of her left side, being able only to describe the pictures localized on the right side of the “cookie theft picture”. She preferred right side gaze spontaneously, but there was neither sustained conjugate eye deviation nor gaze palsy. Extrinsic ocular movements were full on smooth pursuits and hemianopsia was not present. The National Institute of Health Stroke Score^9^ (NIHSS) was 7. Computed tomography (CT) of the brain was normal on initial evaluation, but a conjugated rightward shift of the eyes, or “eyes sign”, was present. (Figure 1). Intravenous rt-PA was administered 180 minutes after the onset of symptoms. The patient made an almost full recovery, remaining only with a mild flattening of the naso-labial fold and asymmetry on smiling (NIHSS of 1). At follow up 24 hours later, brain magnetic resonance imaging (MRI) confirmed an infarct in the right insular subcortical region (subinsular territory) (Figure 2). Despite recovering from hemispatial neglect, the patient remained unaware of her recent stroke and slight neurological deficits (anosognosia). Initially she denied any neurologic problem. After receiving an explanation concerning the illness, she accepted the fact she had suffered a stroke, although this appeared to have had little impact on her behavior. The patient remained free of hemispatial neglect at the follow up visit.

## Discussion

Neglect may be conceptualized as a spatially addressed bias of sensory events in explicit behaviors, in the absence of perceptual or motor deficits. Patients with hemispatial neglect act as if the sensory events that occur in the neglected hemispace had lost their impact on awareness, especially when stimuli from the other side are present simultaneously (extinction). Extinction is characterized by normal responses to unilateral stimulation on either side but neglect of one side (usually the left) under conditions of simultaneous bilateral stimulation. In some patients, the tendency to exhibit extinction is so strong that the mere presence of visual stimulus on the normal side can cause neglect of the other side. Visual scanning is also affected, with impairment in exploratory eye movements in the neglect hemispace, even in the absence of gaze palsy. Akin to other cortical dysfunctions, neglect is also a “network syndrome” in which highly spread components with different functional specialization and anatomical sites are interconnected. The right hemisphere is dominant for the processing of attention. Cortical epicenters of the human “attentional network” are localized in the posterior parietal cortex (the principal subdivisions implicated in neglect are the banks of the intraparietal sulcus followed by superior and inferior parietal lobules and less frequently, medial parietal cortex), frontal eye fields and cingulated gyrus, encoding representational, exploratory and motivational aspects of spatial information, respectively.^7^ Lesions in these areas have been consistently implicated in neglect.

Insular and subinsular lesions have also been associated with neglect.^10,11^ Our patient had a stroke involving the subinsular region. A subinsular infarct is defined as a lesion involving the region running parallel and subjacent to the insular cortex, for at least one-third of the anteroposterior length of the insular cortex. This area involves only the subcortical component of the insula and is typically a border zone between the small insular penetrating arteries and the branches of the lenticulostriate arteries.^11^ In a study involving 11 patients with isolated subinsular strokes,^11^ 2 patients presented with neglect (one with visuo-spatial neglect and 1 with tactile extinction).

Insular cortex has several connections with other cortical areas, including frontal, parietal and limbic regions.^12^ Lesions of the insula may disrupt connections of the structures involved in the control of hemispatial attention.

Alternatively, a recent study has shown that insular cortex, in addition to the superior temporal cortex, putamen and caudate nucleus, are the most frequently damaged neural structures in patients with right hemisphere lesion and spatial neglect compared to patients with right hemisphere lesion without neglect,^13^ providing new insights on the functional neuroanatomy of the “attentional network” in humans. According to this data, lesions in the “multisensory vestibular cortical” areas important in spatial encoding of the surrounding space in terms of body position (including head and body orientation) namely the posterior insula, retroinsular regions, superior temporal gyrus and temporo-parietal junction, seem to correspond anatomically to areas capable of causing neglect.^14^ Deregulation in spatial processing of head and body orientation at a cortical level may induce neglect (a spontaneous bias of eye and head to the right due to left inattention), comparable to the behavior problems presented by patients with unilateral peripheral vestibular dysfunction (a constant deviation of eyes and head to the horizontal plane).^14^ These findings may link vestibular functions to neglect syndromes.

Although conjugated eye deviation has been associated with hemispatial neglect,^4^ while some milder forms may only be evident with eyes closed^2^ (removing gaze fixation), our patient, when assessed for smooth pursuit and lateral eye movements in response to verbal command, had a normal exam devoid of sustained deviation of the eyes, gaze palsy or paresis.

We suppose that following bilateral symmetric stimuli presented during the CT exam, the patient developed deviation of the eyes towards the right side of the space, corresponding to visual extinction. Alternatively, even in the dark or with eyes closed, eye fixation could still be deviated to the right due to biased ocular searching. In one study, patients with visual neglect were evaluated in a completely darkened room yet fixations were confined almost entirely to the right of the midline.^15^ Another study has shown that spontaneously horizontal deviation of the eyes and head are specifically associated with lesions that cause clinical spatial neglect, when measured after 1.5 days (on average).^3^

Neglect can be evaluated by using several types of spatial attention tests. Simple observation is able to identify the most severe forms. Patients may turn their head and eyes to the right and not gaze to the left, may ignore the external word on the left-hand side and may even ignore the left side of their body. However, in most patients identifying neglect is not straight forward. There are several bedside screening instruments available, including object copying tasks, picture description, clock drawing test, word cancellation and line bisection.^16,17^ The inclusion of one simple test (line cancellation test) has improved the assessment of neglect in acute stroke patients.^18^ More complete batteries have been created for evaluating neglect^19^ and have proved to be more sensitive than simple screening tests.^20^ Nevertheless, in acute settings where time is limited, these tests are often unpractical.

In conclusion, patients with right hemisphere stroke are less likely to be identified and treated.^21^ Hemispatial neglect is a common cortical syndrome presenting in acute brain lesions. However, it occasionally goes unrecognized in the emergency department, having repercussions on therapeutic decision-making. This case revealed an early indirect CT “eye sign”, that may be present in patients with hemispacial neglect without conjugate eye deviation. Prompt recognition of this sign may lead to timely identification and assessment of hemispacial neglect in acute stroke patients.
